# Important Considerations in Developing and Evaluating Intergenerational Service-Learning Programs

**DOI:** 10.1093/geroni/igaa057.1768

**Published:** 2020-12-16

**Authors:** Laura Donorfio

**Affiliations:** University of Connecticut, Waterbury, Connecticut, United States

## Abstract

Very often, intergenerational programs measure their success by collecting feedback primarily from the students participating in the program. Critical stakeholders such as the adults/older adults, administrators, and service-learning personnel can be overlooked, as well as the impact of the various classroom activities and tools used. Each fall semester over a five-year period, an undergraduate adulthood and aging intergenerational service-learning course was offered, measuring and building on the successes and challenges from the previous year. No matter how much thought goes into planning a class, unexpected variables naturally unfold over time. This presentation will highlight a best practices model developed over a five-year period with feedback collected from key constituents. Engaging different generations in a purposeful service-learning experience is complex and intended outcomes are not guaranteed. Understanding the key variables and the needs of all those involved through feedback and measurement, can help ensure a more meaningful educational experience. Part of a symposium sponsored by Intergenerational Learning, Research, and Community Engagement Interest Group.

